# Therapeutically Significant MicroRNAs in Primary and Metastatic Brain Malignancies

**DOI:** 10.3390/cancers12092534

**Published:** 2020-09-07

**Authors:** Akilandeswari A. Balachandran, Leon M. Larcher, Suxiang Chen, Rakesh N. Veedu

**Affiliations:** 1 Centre for Molecular Medicine and Innovative Therapeutics, Murdoch University, Murdoch, WA 6150, Australia; Akila.Balachandran@murdoch.edu.au (A.A.B.); Leon.Larcher@murdoch.edu.au (L.M.L.); s.chen@murdoch.edu.au (S.C.); 2 Perron Institute for Neurological and Translational Science, Nedlands, WA 6009, Australia

**Keywords:** miRNA, glioma, glioblastoma, brain cancer, cancer metastasis

## Abstract

**Simple Summary:**

The overall survival of brain cancer patients remains grim, with conventional therapies such as chemotherapy and radiotherapy only providing marginal benefits to patient survival. Cancers are complex, with multiple pathways being dysregulated simultaneously. Non-coding RNAs such as microRNA (miRNAs) are gaining importance due to their potential in regulating a variety of targets implicated in the pathology of cancers. This could be leveraged for the development of targeted and personalized therapies for cancers. Since miRNAs can upregulate and/or downregulate proteins, this review aims to understand the role of these miRNAs in primary and metastatic brain cancers. Here, we discuss the regulatory mechanisms of ten miRNAs that are highly dysregulated in glioblastoma and metastatic brain tumors. This will enable researchers to develop miRNA-based targeted cancer therapies and identify potential prognostic biomarkers.

**Abstract:**

Brain cancer is one among the rare cancers with high mortality rate that affects both children and adults. The most aggressive form of primary brain tumor is glioblastoma. Secondary brain tumors most commonly metastasize from primary cancers of lung, breast, or melanoma. The five-year survival of primary and secondary brain tumors is 34% and 2.4%, respectively. Owing to poor prognosis, tumor heterogeneity, increased tumor relapse, and resistance to therapies, brain cancers have high mortality and poor survival rates compared to other cancers. Early diagnosis, effective targeted treatments, and improved prognosis have the potential to increase the survival rate of patients with primary and secondary brain malignancies. MicroRNAs (miRNAs) are short noncoding RNAs of approximately 18–22 nucleotides that play a significant role in the regulation of multiple genes. With growing interest in the development of miRNA-based therapeutics, it is crucial to understand the differential role of these miRNAs in the given cancer scenario. This review focuses on the differential expression of ten miRNAs (miR-145, miR-31, miR-451, miR-19a, miR-143, miR-125b, miR-328, miR-210, miR-146a, and miR-126) in glioblastoma and brain metastasis. These miRNAs are highly dysregulated in both primary and metastatic brain tumors, which necessitates a better understanding of their role in these cancers. In the context of the tumor microenvironment and the expression of different genes, these miRNAs possess both oncogenic and/or tumor-suppressive roles within the same cancer.

## 1. Introduction

Brain cancers, also referred to as central nervous system (CNS) tumors, are rare cancers that account for significant mortality in both adults and children. The most common adult CNS tumors are meningioma, pituitary tumors, and malignant gliomas, whereas in children, pilocytic astrocytoma, embryonal tumors, and malignant gliomas are more frequent [1]. In this review, we focus on glioblastoma (GBM) and metastatic brain cancers. Glioma can arise from any of the three types of glial cells, i.e., astrocytes, oligodendrocytes, or ependymal cells. GBM is a highly aggressive grade IV astrocytic tumor that corresponds to 16% of primary brain cancers [2], with an overall survival of approximately 15 months. Brain metastasis (BrM) is also an intracranial tumor accounting for up to 10 times the incidence of primary brain tumors, occurring majorly from melanoma, breast, and lung cancers, as well as from kidney, colorectal, prostate, testicular, ovarian cancers, and sarcoma [3]. The lack of effective treatments illustrates the dire need for the development of targeted and specific therapies capable of tackling primary and secondary brain malignancies. Recently, the definition of oncogenes and tumor suppressors has been expanded to non-protein coding genes, including microRNAs (miRNAs), which play a central role in regulating numerous metabolic and cellular pathways responsible for cell proliferation, differentiation, and survival. This review will focus on differential expression of miRNAs in primary and metastatic brain cancers and their potential application in prognosis, diagnosis, and treatments. 

## 2. Brain Cancer

### 2.1. Primary Brain Cancers

Primary brain cancers are heterogeneous tumors that arise from the cells of CNS [4]. Brain tumors are either benign (meningiomas, pituitary tumors, astrocytomas, etc.) or malignant (high-grade astrocytoma, gliomas, etc.) [2]. Primary brain tumors are classified as grade I to IV, according to the World Health Organization (WHO) CNS tumors grading system. The most common malignant primary brain tumors are gliomas, representing approximately 75% in adults [4]. Glioma tumors include astrocytoma, oligodendroglioma, ependymoma, and mixed glioma, of which 50% are GBM. GBM accounts for ~40% of all CNS malignancies and 50% of all primary brain tumors [5]. GBM is further classified into primary and secondary GBM. Primary GBM develops from non-malignant cells, whereas secondary GBM develops from low-grade gliomas. Both primary and secondary GBM have different molecular profiles and hence, require different treatment strategies [6]. Outcomes for GBM patients remain grim, with a 5-year survival rate of less than 10% compared with the 67% overall 5-year survival rate of patients with cancer of other types.

### 2.2. Brain Metastasis

A major cause of cancer death is metastasis. Nearly 40% of cancer patients progress towards BrM, accounting for 50% from lung, 25% from breast, and 20% from melanoma. Additionally, renal cell carcinoma and colorectal cancer (CRC) could also lead to BrM. Therapeutic options available for BrM can be grouped into two categories: symptomatic management and tumor-targeted therapies [7]. The median overall survival of patients with BrM is less than 6 months if treated and 1–2 months without treatment [8].

## 3. miRNA Biogenesis

**Figure 1:**
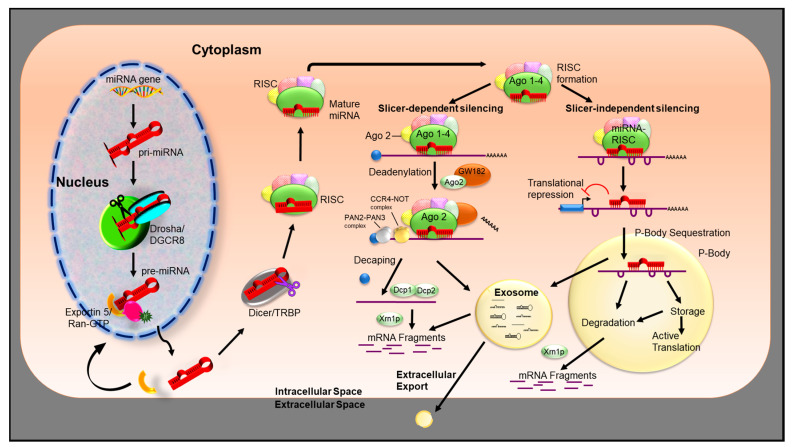
miRNA biogenesis and mechanism of action. miRNAs are transcribed by RNA polymerase II or III, producing primary transcripts (pri-miRNA). The pri-miRNA is then cleaved by the Drosha and DGCR8 complex, producing precursor miRNA (pre-miRNA). Pre-miRNA assembles into a complex with Exportin-5 (XPO5) and Ran/GTP. Once in the cytoplasm, pre-miRNA is further processed by Dicer complex into mature miRNA. The passenger strand of mature miRNA is degraded, while the other strand is loaded into argonaute protein (Ago1–4) and incorporated into the RNA-induced silencing complex (RISC). Binding of RISC to its target mRNA results in degradation and/or translational repression of the target gene, by either slicer-independent or slicer-dependent silencing. When the miRNA is extensively base-paired, slicer-dependent silencing mechanisms proceed. GW182 family of proteins is recruited by AGO. GW182 interacts with PABPC, promoting efficient mRNA deadenylation through the recruitment of PAN2–PAN3 and CCR4–NOT complexes. Cleavage begins with the deadenylation of the mRNA to remove the poly (A) tail. Deadenylation promotes subsequent mRNA decapping and degradation by Xrn1p. Alternatively, degradation can occur via the exosome (vesicles with a size of 30 to 100nm, with 3′→5′ exonuclease activity). In slicer-independent silencing, multiple complementary sites with imperfect base-pairing create bulges in the RNA duplex propelling slicer-independent gene silencing mechanisms. miRNAs can repress translation determined by the target mRNA promoter. Alternatively, miRNA can repress translation indirectly by segregating mRNA into P-Bodies. Ultimately, mRNA can be isolated for storage or be targeted for decay via Xrn1p or exosome degradation.

The biogenesis and mechanism of action of miRNA is illustrated in Figure 1. miRNAs are encoded by nuclear DNA and are typically transcribed by RNA polymerase II or III, producing capped and polyadenylated long primary transcripts, termed primary miRNA (pri-miRNA) [9]. The pri-miRNA folds into a large stem-loop structure via intramolecular base-pairing [10] with single stranded RNA extensions at both ends [11,12,13]. Pri-miRNA is then cleaved by the microprocessor protein complex, consisting of the RNase III endonuclease Drosha and the double-stranded RNA-binding protein DGCR8 (DiGeorge syndrome critical region gene 8) [9,14,15,16]. This process results in the generation of a precursor miRNA (pre-miRNA) hairpin of 60–120 nucleotides in length [9,16]. The pre-miRNA is then assembled into a complex with nucleocytoplasmic transporter factor Exportin-5 (XPO5) and Ran/GTP, preventing nuclease degradation and facilitating its translocation from nucleus to cytoplasm [17,18,19,20,21]. The pre-miRNA undergoes further processing in the cytoplasm by the RNase II enzyme Dicer complex. Dicer cleaves the hairpin loop, producing a ~22 bp miRNA duplex, consisting of a passenger strand and a mature miRNA strand (guide strand).

The association of Dicer with RNA-binding domain factors, protein activator of PKR (PACT), or trans-activation response RNA-binding protein (TRBP), can also produce miRNAs of slightly different sizes, termed isomiRs, which have altered target-binding specificities [22]. Following mature miRNA formation, the duplex is unwound, while the passenger strand is normally degraded; the other mature miRNA strand (guide strand) of the duplex is then loaded onto argonaute protein (Ago1–4) and incorporated into the RNA-induced silencing complex (RISC). Within RISC, the miRNA serves as a template for recognizing its complementary mRNA molecules [23]; miRNA–RISC complex (miRISC) binds target mRNA through Watson–Crick base-pairing between the guide strand and the 3′-untranslated region (UTR) of the target [24,25]. Target recognition relies heavily on base-pairing between the seed sequence (residues 2–8 at the 5′ end) of the miRNA guide [26,27,28,29]. The miRISC may also be found in the nucleus where it is thought to regulate pre-mRNA [30].

Although we describe canonical miRNA biogenesis, it should be noted that miRNAs can also be generated through a number of non-canonical pathways [31]. The non-canonical biogenesis of miRNAs could be broadly categorized as Drosha/DGCR8 independent and Dicer independent pathways. The mirtron pathway [32,33], miRNAs derived from small nuclear RNA (snoRNA) [34], endogenous short hairpin RNAs (endo-shRNA) [35], precursor tRNA [36,37], endogenous small interfering RNA (endo-siRNA) [38], and the tRNaseZ dependent pathway [39] are grouped into the Drosha/DGCR8 independent pathway. The Dicer independent pathways are either argonaute 2 (AGO2)-dependent [40,41] or tRNaseZ-dependent. Biogenesis of some miRNAs referred to as simtrons (splicing-independent mirtron-like miRNAs) are independent of Dicer, DGCR8, exportin-5, or AGO2 [42]. Detailed reviews of non-canonical biogenesis of miRNA could be found elsewhere [31,43,44,45,46].

### miRNA Mechanism of Action

Binding of miRISC to mRNA is determined by the mRNA target site, generally located in the 3′ UTR of mRNAs and possessing strong complementarity to the seed region (nucleotides 2–8 from the 5′ end of the miRNA) [47] crucial for target recognition. Seed interactions involve nucleotides 2–8, 2–7, and 2–6 [48] and can be supplemented by the binding to the MID domain of an adenine in the target mRNA opposite miRNA nucleotide 1 (t1) [49,50,51], or through additional base-pairing nucleotides 13–16 of the miRNA [52]. Binding of miRISC to its target results in degradation and/or translational repression of target transcripts by slicer-independent or slicer-dependent silencing. Slicer-dependent silencing is catalyzed when miRNA (miRISC) is extensively or completely base-paired over regions including the seed and base 10–11 of the guide strand [53,54,55,56,57,58,59], resulting in the recruitment of the GW182 family of proteins by AGO. GW182 interacts with poly(A)-binding protein C (PABPC) [60], promoting efficient mRNA deadenylation, by the recruitment of poly(A)-deadenylation complex 2 (PAN2)–PAN3 and carbon catabolite repressor protein 4 (CCR4)–NOT complexes [60,61,62,63,64,65,66].

Cleavage begins with the deadenylation of the mRNA to remove the poly (A) tail, which is initiated by PAN2/3 and completed by the CCR4–NOT complex [53,67]. Deadenylation promotes subsequent mRNA decapping by the enzymes DCP1 and DCP2 [64], which facilitates 5′ to 3′ degradation by the exoribonuclease Xrn1p [53,68,69]. The interaction between the miRISC and the miRNA response elements (MRE) (complementary sequences on target mRNA) destabilized the association between AGO and the 3′ end of the miRNA, promoting its degradation [70,71]. Alternately, 3′-5′ exonucleolytic degradation can occur via the exosome, a complex of 3′–5′ exonucleases [72]. In slicer-independent silencing, multiple complementary sites with imperfect base-pairing create bulges in the RNA duplex, inhibiting the slicer activity of Ago2 [11,24,47,53,73,74]. miRNA can repress translation directly pre- or post-translation initiation, which is determined by the target mRNA promoter [75]. Inhibition of translation initiation is caused by interfering with the function of eukaryotic initiation factor 4 A-I (eIF4A-I) and eIF4A-II [60,76,77,78]. In many cases, a functional miRNA MRE interaction occurs via the 5′ seed region (nucleotides 2–8) [79,80]. Alternatively, miRNA can repress translation indirectly by segregating mRNA away from ribosomes to cytoplasmic foci (P-bodies) [81,82,83,84,85,86,87]. As stated, miRNAs play key roles in the progression and growth of malignancies and their responses to therapies. However, the selection of miRNAs will vary depending on the desired outcome of the therapy, and the expression patterns of miRNA within the malignancy, due to miRNA network complexity. miRNAs can also be released from the cytoplasm, functioning extracellularly, in cell-to-cell communication in a hormone-like way.

Recently, mRNAs and miRNAs and other non-coding RNAs have been identified in small vesicles termed “exosomes” (extracellular vesicles with a size of 30 to 100 nm) [88]. These miRNAs loaded into exosomes are taken up by neighboring or distant cells, thereby acting as a mechanism of intercellular communication [89]; exosomes also protect miRNAs from degradation by RNases. It has been shown that miRNA contained within exosomes may not be randomly incorporated, and the proportion of miRNA is higher in exosomes than in their parent cells [90,91], with some miRNAs preferentially entering exosomes [92,93]. Additionally, exosomal miRNA expression levels are altered under different physiological conditions [94,95,96], particularly within the cancer microenvironment. Thus, specific incorporation of miRNAs and the exosome cell–cell communication properties indicate that exosomal miRNAs play an important role in disease progression, modulating tumor immunity and the microenvironment, potentially facilitating tumor growth, invasion, metastasis, angiogenesis, and drug resistance [97,98,99]. 

In addition to being loaded into exosomes, microvesicles, or apoptotic bodies [100,101], extracellular miRNAs can be associated with proteins such as high-density lipoprotein (HDL) [102,103], nucleophosmin 1 (NPM1) [104,105,106] or bound by AGO2 protein outside of vesicles [105]. These modes of action protect miRNAs from degradation and improve their extracellular stability [101].

## 4. miRNAs Differentially Regulated in Brain Cancer

Dysregulation of miRNA expression profiles has been associated in most [107], if not all tumors. The specific classification of miRNA as oncogenes or tumor suppressors can be difficult because of intricate temporal and spatial expression patterns [108]. It is not always clear if the altered miRNA patterns are the direct or indirect causes of cancer, or due to secondary changes in cellular phenotypes. Moreover, a single miRNA can regulate multiple targets [109,110], adding an additional layer of complexity. This is coupled with tissue-specific expression [111] that implicates a single miRNA as a tumor suppressor in one context and an oncogene in another [112]. 

**Figure 2:**
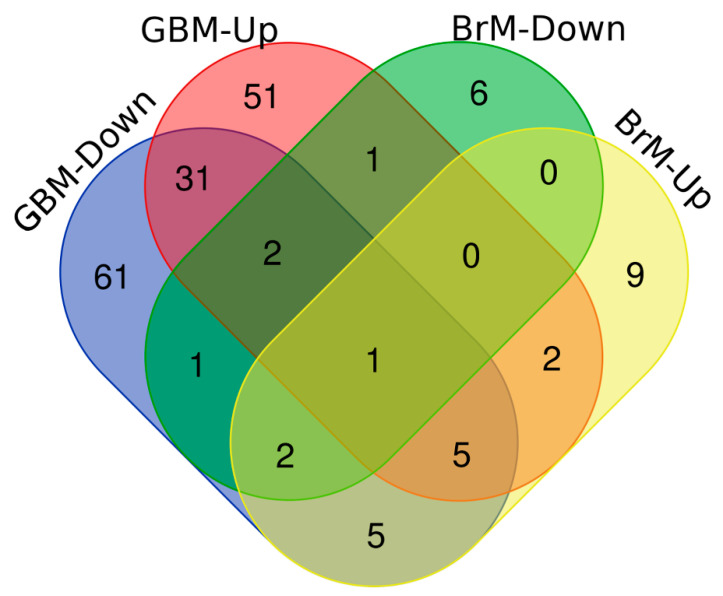
Venn diagram showing the number of miRNAs that are in common between upregulated and downregulated miRNAs in glioblastoma (GBM) and brain metastasis (BrM). Upregulation and downregulation are abbreviated by ‘Up’ and ‘Down’, respectively.

Generally, upregulated miRNAs in tumor cells are considered oncogenic miRNAs (oncomiRs), which can silence tumor suppressor genes. Conversely, miRNAs which are often downregulated in cancers, can inhibit tumor progression and are termed tumor suppressor miRs. These miRNAs target mRNA of some oncogenes and inhibit the carcinogenic effect by repressing translation. miRNA signatures have not only been shown to be dysregulated in cancers; interestingly, restoration of these dysregulated miRNAs have also been shown to abrogate and even reverse the malignant phenotype of cancers [113,114]. Moreover, it has been shown that miRNAs contribute to responses to drug therapy and are themselves modified by drug therapy as well [115,116]. To identify candidate miRNAs of increased therapeutic importance, both upregulated and downregulated miRNAs in primary and metastatic brain cancers were compared. Based on the existing literature, differentially expressed miRNAs in GBM [117,118,119,120,121,122,123,124,125,126,127,128,129] and BrM [130,131,132,133,134,135,136,137] were grouped, which resulted in four different groups, including GBM upregulated, GBM downregulated, BrM upregulated, and BrM downregulated. Venn diagram-based comparison of miRNAs was then performed to identify the putative miRNAs of therapeutic importance (Figure 2 and Table 1), and differentially expressed miRNAs in both GBM and BrM are discussed in detail below. 

In line with recently published reports [132,138,139,140,141,142,143,144], the most important miRNAs implicated in the pathology of GBM and BrM are found to be miR-145, miR-31, miR-451, miR-19a, miR-143, miR-125b, miR-328, miR-210, miR-146a, and miR-126. Modulated expression of these miRNAs has a significant effect on patient survival. Reduced expression of miR-143 [145], miR-328 [146], and miR-126 [147] and increased expression of miR-451 [148], miR-19a [149], and miR-210 [150] in GBM correlates with short life span. Both increased and decreased expression of miRNAs such as miR-145 [151,152], miR-31 [153,154], miR-125b [155,156], and miR-146a [151,157] were reported to contribute to poor survival of GBM patients. In addition, low levels of miR-31 [139], miR-126 [158], and high levels of miR-328 [159] shortened survival of BrM patients. Recent findings on each of these miRNAs are described in the following sections. 

### 4.1. miR-145

MicroRNA-145 (miR-145), located on chromosome 5, is a tumor suppressor miRNA consistently downregulated in most cancers. Reduced levels of miR-145 can be detected in most body fluids, which ensures that miR-145 has a potential role in cancer diagnostics, prognostics, and therapeutics. A detailed review of miR-145 and its mechanisms in cancer is available elsewhere [160,161]. GBM tumors display lower expression of miR-145 and have a direct correlation with overall and progression-free survival of GBM and low-grade glioma patients, respectively [162]. Screening of the pre-miRNA library in GBM, astroglial cell lines, and tumors revealed that miR-145 is consistently downregulated [163]. 

A recombinant adenoviral vector encoding ribozyme targeting telomerase reverse transcriptase (hTERT) and ribozyme-driven herpes simplex virus thymidine kinase (HSVtk) has high anticancer activity and possess dose-dependent cytotoxicity in GBM cell lines. They were reported that increasing miR-145 levels enhance the antitumor activity of HSVtk in GBM [164]. In addition, miR-145 shows tumor-suppressing activity by inhibiting several genes, including *c-Myc*, *IRS1*, *MUC1*, and *FASCN1* [164]. Yang et al. showed that polyurethane-short branch polyethylenimine (PU-PEI) mediated delivery of miR-145 in CD133^+ve^ GBM cells and sensitized them towards chemotherapeutic agents. This PU-PEI-miR145 is also capable of improving the survival of immunocompromised mice with orthotopic CD133^+ve^ GBM tumors. The expression levels of stemness markers SOX2, Oct4, Nanog, Klf4, and Bmi-1 were significantly decreased upon miR-145 overexpression [165]. In GBM neurospheres, miR-145 overexpression showed reduced migration and invasion properties. This also decreased the expression levels of neural precursor cells expressing developmentally downregulated protein 9 (NEDD9), both in vitro and in vivo. NEDD9 contributes to invasiveness of glioma and is directly correlated with tumor grade [162]. In another study, glioma cells transfected with miR-145 showed a reduction in connective tissue growth factor (CTGF), which is involved in the production of extracellular matrix, cell proliferation, invasion, angiogenesis, and migration. CTGF is highly expressed in gliomas, and its expression level correlates positively with tumor progression, migration, and therapy resistance. miR-145 binds to 3’ UTR of CTGF, thereby downregulating its expression, resulting in reduced invasion and migration of glioma cells [166].

To understand the effect of sunitinib and miR-145 mimic in combination, GBM cells were transfected with miR-145 mimic and subjected to sunitinib. Antitumor activity was improved when the cells were subjected to miR-145 mimic and sunitinib combination treatment. Transfection with miR-145 reduced the expression of P-glycoprotein (P-gp) and breast cancer resistance protein (BCRP), resulting in increased distribution of sunitinib leading to improved anticancer activity [167]. Isorhapontigenin (ISO) is a methoxylated derivative of resveratrol, which exerts cell cycle arrest in GBM through modulating the expression of miR-145. By upregulating miR-145 expression, cyclin-D1 and SOX2 are downregulated, leading to G0/G1 arrest in patient-derived glioblastoma spheres (PDGS) resulting in anchorage-independent growth inhibition [168]. Kurogi et al. showed that rat brain slice cultures implanted with U87 and human bone marrow-derived mesenchymal stem cells (hMSCs) showed substantial decrease in invasion [169]. hMSCs possess tropism towards GBM tumors, and these hMSCs designed to co-express and secrete miR-145 and miR-31 via exosomes, possibly repressed the invasiveness of GBM cells. 

Despite these observations, miR-145 also promotes invasive characteristics of glioma. A highly invasive subpopulation of glioma cells isolated from different cell lines was subjected to miRNA microarray analysis and compared with their respective parental cell lines. Among them, miR-143 and miR-145 were significantly upregulated. Downregulating the expression of both miRNAs using respective antisense sequences reduced the invasive phenotypes of all cell lines [170]. Further investigation showed that miR-145 is involved in the SLIT-Robo signaling pathway. SLIT-ROBO Rho GTPase-activating protein1 (srGAP1) is a direct target of miR-145 and is downregulated in invasive cell lines. By regulating srGAP1 expression, miR-145 promotes invasion of glioma cells [171].

Microarray analysis of primary and BrM lung adenocarcinoma tumors revealed decreased expression of miR-145, which was validated in 35 primary and 8 BrM lung adenocarcinoma tumors. This study revealed that overexpression of miR-145 affects the invasion and migration of BrM lung cancer cells but not primary lung cancer cells. Similarly, it has null effect on lymph node metastatic lung cancer cells. This indicates that miR-145 overexpression has significant antitumor activity after lung cancer metastasizing to the brain but not during initiation of metastasis [172]. A comparative analysis of miRNA profiles of normal lung tissue, primary non-small cell lung cancer (NSCLC), BrM of lung cancer, melanoma, breast cancer, and normal brain tissue revealed that miR-145 is significantly downregulated in BrM of NSCLC. This upregulates expression of OCT-4, MUC-1, EGFR, c-MYC, and TPD52 in both primary and BrM lung cancer. By inhibiting methylation of miR-145 locus, miR-145 expression is restored, leading to downregulation of proteins that contribute majorly to migration, invasion, chemo, and radio resistance of cancer cells [138]. miRNA expression profiling of primary and BrM CRC revealed that miR-145 is upregulated in BrM CRC [132]. There is no existing literature that validates the overexpression of miR-145 in BrM CRC.

### 4.2. miR-31

MicroRNA-31 (miR-31) is located on chromosome 9 at p21.3. miR-31 expression is lower in GBM tumors when compared to healthy brain tissues. When overexpressed, this miRNA repressed invasiveness and the migratory potential of GBM cells by regulating the expression of EMP1, RGS4, and TGFBR1. It was also observed that radixin (RDX) is a direct target of miR-31 [173]. miR-31 downregulation and RDX upregulation is correlated with advanced disease pathology and poor overall survival, serving as a prognostic indicator in glioma progression, specifically in higher grade tumors [154]. This was further validated using GBM cell lines and a patient-derived GBM xenograft cell line; it was observed that miR-31 targets TNF receptor-associated death domain (TRADD), thereby reducing NF-κB activation in GBM [174].

Signal transducer and activator of transcription 3 (STAT3) remains constantly activated in GBM by NF-κB signaling, leading to temozolomide (TMZ) resistance. GBM cells transfected with miR-31 showed reduction in STAT3 phosphorylation, affecting the expression of survivin, cyclin D1, and Mcl-1. Together, this reduced cell proliferation and resulted in mitochondrial apoptosis of GBM cells. Elevated expression of miR-31 also increased the cytotoxic activity of TMZ in GBM [175]. In another study, the effect of miR-31 overexpression downregulating the Dock protein Dock1 (Dock180) was investigated. Dock1 has a significant effect on chemotaxis and migratory potential of cancer cells including glioma. miR-31 directly targets 3′ UTR of Dock1 resulting in its deregulation. Since miR-31 is hypermethylated in glioma, Dock1 is overexpressed, leading to IL-8 induced mesenchymal transition. Overexpression of miR-31 in vivo results in Dock1 downregulation followed by reduced invasion of glioma cells. Additionally, DNA methyltransferase inhibitor 5-aza-2′-deoxycytidine potentially increases miR-31 levels, inhibiting invasion of glioma cell lines [176]. Co-expression of miR-145 and miR-31 in human mesenchymal stem cells cultured with GBM cells reduced invasiveness of GBM cells in a contact-dependent fashion. This also showed a decrease in the fascin actin-bundling protein 1 (FSCN1), which led to reduced GBM invasion. [169].

The long noncoding RNA (lncRNA) Forkhead box D2 (FOXD2) adjacent opposite strand RNA 1 (FOXD2-AS1) is overexpressed in glioma tumors and correlates positively with tumor grade. The cancer genome atlas (TCGA) analysis revealed that FOXD2-AS1 is associated with cell cycle regulatory pathways in glioma and GBM. FOXD2-AS1 acts as a competing endogenous RNA (ceRNA) and sponges miR-31. This miR-31 sponging results in the overexpression of cyclin-dependent kinase 1 (CDK1), leading to glioma progression. By silencing FOXD2-AS1, cell cycle arrest and CDK1 downregulation were achieved in glioma cells. The FOXD2-AS1/miR-31/CDK1 axis has better therapeutic potential in glioma cells [177]. Another lncRNA, LINC01116, functions as ceRNA for miR-31, thereby regulating VEGFA expression. In glioma tissues, LINC01116 expression is increased compared to normal brain tissues and is associated with tumor recurrence. Silencing of LINC01116 reduced VEGFA expression and inhibited invasion, migration, angiogenesis, and induced cell cycle arrest of glioma cells both in vitro and in vivo [178]. From these studies, it is evident that miR-31 has a negative effect on glioma progression.

On the other hand, TCGA analysis prediction revealed that miR-31 is associated with poor survival of GBM patients by enhancing pathways related to tumor growth. The increased expression of miR-31 is observed only in a small fraction of GBM tumors. Anti-miR-31-mediated downregulation of miR-31 reduced tumor growth and increased survival of orthotopic GBM xenograft models. These experiments showed that miR-31 targets the factor inhibiting hypoxia-inducible factor 1 (HIF-1) and facilitates hypoxia-inducible factor HIF-1α and Notch signaling pathways. This miRNA has a significant role in the maintenance of stemness in GBM tumors with high expression of miR-31 [179].

miR-31 expression has been identified to be downregulated in BrM CRC, whereas it is upregulated in primary colon cancer [132,133]. Treatment response of metastatic CRC patients undergoing anti-EGFR treatment was predicted using miR-31 expression. This study revealed that patients with lower miR-31 expression had better response to cetuximab treatment [139].

### 4.3. miR-451

In humans, miR-451 is located close to miR-144 on chromosome 17 and has a potential role in the diagnosis, prognosis, and treatment of different cancer types [180]. MicroRNA profiling of GBM, astrocytoma cell lines, and normal brain tissues revealed decreased expression of miR-451 in GBM [181].

Overexpression of miR-451 in GBM cells leads to reduced cell proliferation, invasion, and drug resistance. Gal et al. performed miRNA profiling of CD133^+ve^ and CD133^−ve^ GBM primary tumor cells and showed that miR-451 is upregulated in CD133^−ve^ cells. Overexpression of miR-451 actively reduced neurosphere formation and cell proliferation. An in-depth analysis deciphered that miR-451 is activated via the SMAD pathway. SMAD-mediated upregulation of miR-451 resulted in differentiation and reduced the tumorigenicity of GBM tumor cells [182]. Another study revealed that increasing the expression of miR-451 using 2´-O-methyl (2´-OMe)-modified miR-451 mimics led to apoptosis, reduced viability, and invasion of GBM cells. It was also observed that miR-451 regulates the expression of multiple genes associated with cell cycle progression, cell proliferation, adhesion, and apoptosis through the Akt signaling pathway. In particular, miR-451 regulates *cyclin D1*, *p27*, *MMP-2*, *MMP-9*, and *Bcl-2* [181]. Studies by Alural et al. showed that the acquired resistance and survival of GBM upon erythropoietin (EPO) treatment is mediated by MMP-2, MMP-9, VEGF, C-X-C chemokine receptor type 4 (CXCR4), Bcl-2, survivin, and Akt. By overexpressing miR-451, it is possible to reverse the EPO-mediated resistance acquired by GBM cells [183]. 

miR-451 regulates the 5′ AMP-activated protein kinase complex (AMPK) pathway by targeting liver kinase B1 (LKB1). There exists a feedback loop mechanism between AMPK and miR-451, which could be utilized to improve the efficiency of GBM therapy. Godlewski et al. showed that reduced glucose levels induce AMPK phosphorylation by LKB1. An increase in glucose levels leads to an increase in miR-451, increasing proliferation, and reducing migration. On the other hand, a decrease in glucose levels decreases miR-451 and increases AMPK, decreasing proliferation, and promotes cancer cell migration [148,184,185].

Clear cell renal cell carcinoma (ccRCC) is a primary urological malignancy which, upon gaining metastatic potential, has a very poor prognosis [140]. A study by Heinzelmann et al. revealed that miR-451 expression is increased in primary ccRCC in comparison with BrM ccRCC. The decreased expression of miR-451 upon gaining metastatic potential in ccRCC aligns with the earlier report stating that miR-451 decreases migration and invasion of cancer cells [144]. This shows that miR-451 could serve as a prognostic marker for brain metastasis of ccRCC. 

### 4.4. miR-19a

MicroRNA-19a (miR-19a) located on chromosome 13q31.3, is an oncomiR that belongs to the 17-92 cluster and positively correlates with GBM tumor grade. In patients with progressive glioma, differential expression of 157 miRNAs was analyzed. This revealed that miR-19a is upregulated in glioma progression and inhibition of miR-19a reduced glioma cell proliferation [186]. 

In glioma, miR-19a negatively regulates the expression of tumor suppressor proteins RhoB and RUNX3. Chen et al. reported that miR-19a promoted proliferation, migration, and invasion of glioma cells by downregulating RhoB [187]. Another study showed that miR-19a/b inhibition in vitro and in vivo GBM models resulted in repression of glioma cell proliferation partially by upregulating RUNX3, followed by blocking of the Wnt/β-catenin pathway. Additionally, miR-19a/b inhibition leads to reduced expression of cyclin-D1, c-MYC, AKT1, and VEGF [188].

Phosphatase and tensin homolog (PTEN) is a direct target of miR-19a/b. By downregulating miR-19a, PTEN is upregulated, which leads to reduced invasion, migration, proliferation of glioma cells, and drives glioma cells towards apoptosis and cell cycle arrest. Jia et al. reported that introduction of anti-miR-19a/b in the glioma cell line downregulated miR-19a/b and upregulated PTEN [189]. Another study showed that miR-19a/b and five other miRNAs regulate PTEN expression in irradiated GBM stem-like cells (GSCs). These miRNAs inhibited expression of PTEN in 60-Gy-irradiated GSCs, resulting in reduced inhibition towards cell proliferation, differentiation, and replication [190]. Increasing the expression of lncRNAs that possess miR-19a binding sites inhibits GBM cell proliferation. Studies showed that lncRNAs maternally expressed gene 3 (MEG3) and AC016405.3 overexpression led to miR-19a sponging, thereby increasing PTEN and reducing ten-eleven translocation methylcytosine dioxygenase 2 (TET2), respectively [191,192].

In BrM, miR-19a is consistently upregulated. Zhang et al. showed that in in vivo breast cancer BrM models, PTEN loss is mediated by astrocyte-derived exosomes. Reduction in PTEN expression increased the secretion of C-C motif chemokine ligand 2 (CCL2), resulting in ionized calcium-binding adapter molecule 1 (IBA1) expressing myeloid cell infiltration in the BrM tumor. When astrocytes were deprived of PTEN targeting miRNAs (miR-19a) or inhibited by exosome secretion, PTEN expression was restored, and BrM was suppressed [193]. Another study reported that miR-19a is overexpressed in primary and BrM prostate cancer cell lines, but downregulated in bone and lymph node metastatic prostate cancer cell lines [141]. This shows that miR-19a is differentially expressed in different metastatic sites within the same cancer.

### 4.5. miR-143

MicroRNA-143 (miR-143) is located at chromosome 5q32 and belongs to the miR-143/145 cluster; additionally, miR-143 is differentially expressed in cancers [194]. In GBM, miR-143 expression is significantly reduced and is inversely correlated to tumor grade. NUAK family SNF 1-like kinase 2 (NUAK 2) is a protein kinase overexpressed in glioma cells and correlates positively with glioma growth and progression. Fu et al. reported that NUAK2 is a direct target of miR-143, which is involved in the maintenance of GSCs by upregulating EZH2, CD133, STAT-3, Bmi-1, MDR1, and SSEA-1. By increasing the levels of miR-143, NUAK2 is downregulated, resulting in decreased proliferation, invasion, and migration of GBM cells [195].

A combinatorial treatment of restoring miR-143 expression and chemotherapy reduces GSC stemness and induces apoptosis in GBM cells both in vitro and in vivo. miR-143 negatively regulates hexokinase 2 (HK2), neuroblastoma RAS viral oncogene homolog (N-RAS), and Bcl-2-associated athanogene 3 (BAG3). Zhao et al. reported that increasing the expression of miR-143 in GSCs altered the expression levels of HK2 and glycolysis. When glycolysis inhibitor 2-Deoxy-D-glucose (2-DG) was added in combination with miR-143, the activity of 2-DG was improved [196]. Another study showed that overexpression of miR-143 reduced N-RAS expression, leading to inhibition of PI3K/AKT, extracellular signal-regulated kinase (ERK1/2) phosphorylation, and P65 accumulation in the nucleus. This resulted in reduced tumor growth, invasion, migration, and angiogenesis in GBM cells. Additionally, miR-143 overexpression in combination with TMZ treatment increased the apoptosis of GBM cells [197]. Liu et al. observed that when GSCs are subjected to lower concentrations of shikonin, the expression of BAG3 is increased, leading to drug resistance. miR-143 levels were also downregulated in these shikonin-treated GSCs. By overexpressing miR-143 in shikonin-treated GSCs, BAG3 expression was reduced leading to apoptosis [198]. 

Despite these findings, the oncogenic role of miR-143 in GBM is also evidenced. Microarray analysis and real-time PCR revealed that miR-143 is overexpressed in formalin-fixed paraffin-embedded (FFPE) GBM tumors and contributes to the invasiveness of highly invasive GBM cell lines. Koo et al. reported that inhibition of miR-143 and miR-145 expression reduced the invasive properties of highly invasive GBM cell lines [170]. Another study revealed that miR-143 inhibition led to reduced proliferation, increased apoptosis, and cell cycle arrest. Injection of miR-143 inhibitor into tumor bearing mice showed a decrease in tumor growth. This study also led to the finding that miR-143 targets solute carrier family 30 (zinc transporter) member 8 (SLC30A8), a protein involved in glucose metabolism [199].

In secondary brain tumors, miR-143 is found to be upregulated in CRC BrM. Microarray analysis of primary and BrM CRC samples revealed that miR-143 was upregulated in BrM [132]. There is no other evidence in the literature that substantiates the expression of miR-143 in BrM.

### 4.6. miR-125b

MicroRNA-125b (miR-125b) is located on chromosome 11q23.1 and is uniformly distributed in astrocytes and neurons [200]. miR-125b plays a significant role in cell proliferation and neuronal differentiation. miR-125b is overexpressed in primary and recurrent GBM tumors, and GSCs, and corresponds to TMZ resistance. Xia et al. showed that miR-125b expression was reduced when glioma cells were treated with all-trans-retinoic acid (ATRA). Decreased expression of miR-125b resulted in reduced cell proliferation and ATRA-induced apoptosis in glioma cells. miR-125b directly binds and negatively regulates the Bcl-2 modifying factor (*Bmf*), thereby sensitizing these cells to apoptosis [201]. The combination of miR-125b inhibitors with TMZ increased apoptosis of GBM cells. This has been studied by several groups, and the mechanisms reported are compiled. The combination therapy increased apoptosis via shift in Bax/Bcl2 ratio, increased cytochrome c (Cyt c) levels, caspase 3 activation, and expression of APaf-1 and poly-ADP-ribose polymerase (PARP) [202]. It also reduced proliferation and invasion via Notch 1 downregulation. In addition, combinatorial use of inhibitors for both miR-125b and PI3K resulted in inactivation of Wnt/β-catenin signaling [203], by downregulation of Connexin43 (CX43) [204]. This treatment sensitized cells to TMZ via Bak1 restoration (upregulation) [205], inactivation of Wnt/β-catenin signaling [203], and Notch 1 downregulation, which was achieved via protein inhibitor of activated STAT (PIAS3). PIAS3 negatively regulated STAT3, MMP2, and MMP9, hence reduced invasion, and sensitized GSCs towards TMZ [206]. 

TMZ resistance in GBM is often correlated with O6-methylguanine-DNA methyltransferase (MGMT), NF-κB, and tumor protein 53 (TP53). Haemmig et al. reported that miR-125b mediated NF-κB activity by downregulating tumor necrosis factor alpha-induced protein 3 (TNFAIP3) and NF-κB inhibitor interacting RAS-like 2 (NKIRAS2), resulting in TMZ resistance in GBM cells. The TMZ resistance observed is independent of MGMT and TP53-associated chemoresistance, suggesting that miR-125b levels could be used for prognosis of TMZ resistance in GBM cells [207]. Additionally, Shi et al. reported that miR-125b correlates positively with the migration of CD133^+ve^ GSCs, and miR-125b regulates the expression of matrix metalloproteins (MMP-2, MMP-9) and its inhibitors (RECK and TIMP3), modulating the invasiveness of CD133^+ve^ GSCs [208]. Moreover, Wan et al. stated that increased levels of MMP2, MMP9, and Ki-67 accompanied by increased expression of miR-125b in SU3 cells increased its invasiveness both in vitro and in vivo [209]. 

On the other hand, miR-125b expression was decreased in three primary CD133^+ve^ GSCs and regulated proteins involved in the cell cycle. Shi et al. found that increasing miR-125b levels in CD133^+ve^ GSC leads to the blockade in G1/S transition via downregulation of CDK6 and CDC25A [210]. Another study revealed that G1 phase arrest was mediated by targeting the E2F2 transcription factor [211]. Wan et al. reported that overexpression of miR-125b led to inhibition of GSCs proliferation by arresting them in the G0/G1 phase. It was also observed that Lin28 is negatively regulated by miR-125, thereby its overexpression reduced Lin28 levels, leading to decreased GSC proliferation [212]. Myc-associated zinc finger protein (MAZ) is a transcription factor that regulates the expression of several genes and is inversely correlated to miR-125b. Smits et al. reported that miR-125b is downregulated in patient-derived GBM-associated endothelial cells and normal brain endothelial cells exposed to GBM conditioned media. There exists a feed-forward loop in angiogenesis of GBM where miR-125b expression is hindered by VEGF, leading to an increase in MAZ, in turn, increasing VEGF. By interfering MAZ/VEGF/miR-125b loop, angiogenesis of GBM can be abrogated [213]. Li et al. studied the prognostic and predictive values of miRNAs in glioma and stated that miR-125b is a potential marker that could differentiate cancerous and healthy tissues. Increased expression of miR-125b is directly correlated with advanced glioma tumors and poor prognosis [214]. In MGMT unmethylated GBM, miR-125b is reported to be associated with poor prognosis [155]. Likewise, Jesionek-Kupnicka et al. correlated miRNA expression with TP53 and MGMT expression in GBM and suggested that miR-125b negatively correlated with MGMT levels in MGMT methylated patients [215].

miRNA profiling of cerebrospinal fluid (CSF) revealed that miR-125b is upregulated in BrM. Drusco et al. reported that miR-125b is significantly upregulated in CSF of primary brain cancers and breast and lung metastatic brain cancers, when compared to non-cancerous CSF samples [216].

### 4.7. miR-328

MicroRNA-328 (miR-328), a tumor suppressor miRNA located on chromosome 16q22.1 [186], negatively regulates ATP-binding cassette subfamily G member 2 (ABCG2), thereby playing a vital role in drug disposition in cancer cells [217]. In GBM, miR-328 is inversely correlated with tumor grade and its expression decreases with tumor progression [186]. Wu et al. performed microarray analysis of GBM tumors and revealed that miR-328 is consistently downregulated. It was also evident that miR-328 expression correlates negatively with oncogenes such as *KIF23* and *E2F1* [146]. 

In contrast, high-grade infiltrating gliomas show increased expression of miR-328. Secreted frizzled-related protein 1 (SFRP1) is hypermethylated due to overexpression of miR-328, leading to the activation of Wnt signaling in infiltrative gliomas. Delic et al. revealed that miR-328 facilitated Wnt signaling activation, resulting in increased proliferation and invasion of glioma cells. However, the expression of miR-328 in primary GBM is similar to that of non-tumor brain tissues. This observation suggests that miR-328 levels in low-grade gliomas are of high importance in contrast with high-grade gliomas, since this correlates with the invasiveness of the tumor and SFRP1 mediated Wnt activation [218]. 

Microarray analysis of BrM and non-BrM NSCLCpatient samples revealed that miR-328 and miR-330-3p could potentially discriminate BrM and non-BrM NSCLC. Arora et al. reported that expression of miR-328 is significantly upregulated in BrM NSCLC, in both primary and metastatic tumor samples. This observation was further validated in a larger cohort of 86 BrM and non-BrM samples, where miR-328 was significantly altered between the groups and in adjacent tissues of the BrM group. Additionally, miR-328 regulated expression of protein kinase C-alpha (PRKCA), interleukin 1 beta (IL-1beta), c-Raf, and KCNMA1 that are involved in metastasis [219]. Another study evidenced that PRCKA expression is miR-328-dependent and plays an important role in BrM of NSCLC [159]. These results suggest that miR-328 could potentially be used as a prognostic marker of BrM in NSCLC.

### 4.8. miR-210 

The master hypoxamiR, miR-210, is located on chromosome 11p15.5 [220]. miR-210 expression is consistently upregulated in hypoxic environments and plays essential roles in cell proliferation, apoptosis, angiogenesis, mitochondrial metabolism, and response to DNA damage. miR-210 is significantly upregulated in GBM tumors and cell lines, and is associated with poor prognosis [221]. This miRNA directly targets regulator of differentiation 1 (ROD1) and SIN3 transcription regulator family member A (SIN3A), and is regulated by hypoxia-inducible factor 1-alpha (HIF-1α) and SIN3A.

In the GBM microenvironment, hypoxia is a dominant feature and is related to aggressive tumor phenotypes. Rosenberg et al. reported a direct correlation between hypoxia and miR-210 levels, thus validating the HIF-1α/miR-210 regulatory mechanism in hypoxic conditions [222]. In GBM, inhibition of miR-210 led to decreased proliferation, invasion, and increased apoptosis, possibly through targeting ROD1 and SIN3A. A study by Zhang et al. showed that miR-210 negatively regulates the repressive splicing regulator ROD1. Inhibitors of miR-210 substantially increased ROD1 expression, resulting in apoptosis and cell proliferation inhibition [223]. Another study revealed that SIN3A, a transcriptional repressor, is downregulated in glioma, and its expression is negatively correlated with miR-210. By regulating SIN3A, miR-210 increased the survival and proliferation of glioma cells [224].

Interestingly, miR-210 was observed to be downregulated in GBM and is correlated with poor prognosis. Overexpression of miR-210 decreased invasion and migration of GBM cells by regulating prolyl 4-hydroxylase beta polypeptide (P4HB) and brain-derived neurotrophic factor (BDNF). Lee et al. reported that miR-210 is significantly reduced in TMZ-resistant GBM cells. It was observed that overexpression of miR-210 reversed TMZ resistance via P4HB downregulation [225]. Another study explained that increased BDNF expression is associated with initiation and progression of GBM. There exists a negative correlation between miR-210 and BDNF. Overexpression of miR-210 reduced invasion and migration of GBM cells [226].

Small RNA sequencing of primary and BrM lung adenocarcinoma (LAC) tumors revealed that miR-210 overexpression is correlated with BrM of LAC and high risk of tumor relapse. Daugaard et al. validated miR-210 expression in metastatic and non-metastatic LAC and concluded that upregulation of miR-210 is clearly associated with LAC BrM [143].

### 4.9. miR-146a

MicroRNA-146a (miR-146a) is located on chromosome 5q33.3 in humans and chromosome 11 (band B1.1) in mice. This miRNA is associated with several pathologies, including cancer, inflammation, innate immune response, and cardiovascular and kidney diseases [227]. miR-146a is reported to be upregulated in melanoma, multiple myeloma, bladder, and cervical cancers, downregulated in GBM, myeloid malignancies, prostate, pancreatic, ovarian, and esophageal cancers, and differentially regulated in liver, breast, thyroid, oral, gastric, colorectal, and NSCLC [228]. Mei et al. reported that overexpression of miR-146a in malignant murine astrocytes and human GBM cells inhibited tumor growth by downregulating neural stem cell factor Notch1. This resulted in differentiation of neural stem cells, preventing the formation of GSCs and metastasis. In addition to the Notch pathway, miR-146a regulates PTEN, EGFR, and NF-κB signaling pathways [229]. 

A study by Permuth-Wey et al. showed that rs2910164 polymorphism in precursor miR-146a resulted in reduced levels of mature miR-146a. This, in turn, modulated the regulation of tumor necrosis receptor-associated factor 6 (TRAF6) and interleukin-1 receptor-associated kinase 1 (IRAK1). Specifically, rs2910164 CC/GC genotypes were reported to be associated with increased risk of glioma in elderly people and the C allele with decreased survival of GBM patients [157]. Liu et al. reported that rs2910164 CC genotype is linked to decreased survival of high-grade glioma patients with a gradual decrease in mature miR-146a expression. A similar trend was found in low-grade gliomas with no significant effect on survival rate. Notch1 and Notch2 are differentially regulated by miR-146a, confirmed by the introduction of miR-146a mimics resulting in gradual decrease in Notch1/Notch2 ratio and vice versa. Loss of miR-146a due to the rs2910164 polymorphism altered the Notch1/Notch2 ratio, leading to malignant transformation of GBM cells [230]. Another study also revealed that miR-146a is significantly downregulated in GBM tumor tissues compared to non-tumor tissues. By increasing miR-146a expression, Notch1 is downregulated, leading to increased apoptosis in GBM cells [231].

The resistance of GBM to TMZ therapy is facilitated by NF-κB signaling. Wu et al. showed that combinatorial use of curcumin (a negative regulator of NF-κB) and TMZ treatment is effective in GBM cells. Difluorinated curcumin upregulated the expression of miR-146a, which, in turn, inhibited NF-κB signaling, thereby sensitizing GBM cells towards TMZ induced apoptosis [232].

In metastatic cancers, miR-146a expression is differentially regulated. Hwang et al. studied the xenograft models of brain-trophic metastasis and revealed that miR-146a expression was lost in metastatic melanoma cells. Overexpression of miR-146a resulted in a reduction in migration and invasiveness in metastatic cells. Additionally, miR-146a inhibits metastatic activity of BrM melanoma cells by upregulating β-catenin and downregulating hnRNPC [233]. In contrast, microarray analysis done by Li et al. revealed that miR-146a is upregulated in BrM CRC compared to primary CRC [132].

### 4.10. miR-126

MicroRNA-126 (miR-126) is located on chromosome 9q34.3 and is highly expressed in vascularized tissues [234]. Reduced expression of miR-126 was observed in different cancers, indicating that miR-126 is a potential tumor suppressor. Han et al. revealed that miR-126 is significantly downregulated in GBM. Among the samples, high-grade GBM exhibited the lowest expression of miR-126 compared to low-grade tumors. Additionally, the survival of these patients was correlated with intratumoral expression levels of miR-126 [235]. Another study investigated the methylation of miR-126 in glioma tumors and showed that the extent of methylation is associated with tumor grade. This study confirmed that reduced expression of miR-126 leads to gliomagenesis and progression [236]. 

miR-126 negatively regulates insulin receptor substrate 1 (IRS-1), Kristen rat sarcoma viral oncogene (KRAS), and GATA binding protein 4 (GATA4) and its loss is implicated in tumor progression. Luan et al. showed that IRS-1 expression is upregulated in several cancer cells compared with the adjacent non-cancerous cells and is reported to play a major role in tumorigenesis and metastasis. IRS-1 is regulated by miR-126 through the PI3K/AKT signaling pathway. Increasing miR-126 expression resulted in reduced proliferation, migration, and invasiveness and promoted cell cycle arrest and apoptosis in cancer cells both in vitro and in vivo [237]. Another study showed that in glioma, decreased expression of miR-126 leads to increased expression of KRAS, resulting in aberrant ERK signaling and promotion of proliferation and invasion. Increasing miR-126 expression in glioma potentially reduced ERK signaling, reversing this effect [238]. Xu et al. reported that transcription factor GATA4 is overexpressed, and it regulates cytoskeletal reorganization, thereby promoting motility and migration of cells. Overexpression of miR-126 results in decreased GATA4 expression, thus, regulates antimetastatic activity of GBM [239].

In GBM, miR-126 regulates different signaling pathways. Chen et al. observed that overexpression of miR-126 lead to PI3K, p-Akt, and MDM2 protein downregulation and PTEN and P53 protein upregulation. This indicated that miR-126 regulates cell proliferation and apoptosis through PTEN/PI3K/Akt and MDM2/P53 signaling pathways [147]. Another study conducted by Cui et al. revealed that miR-126 could potentially induce TMZ sensitivity and overcome resistance in GBM cells, by downregulating the expression of SOX2 (oncoprotein and associated with drug resistance in cancer) and Wnt/β catenin signaling. This suggested that miR-126 TMZ combination therapy might be a potential therapeutic strategy for GBM [240].

Tavazoie et al. studied the expression of miR-126 in primary and lung, bone, or brain metastasized breast tumors. This revealed that loss of miR-126 is associated with breast cancer relapse and metastasis. It was evident that miR-126 is a metastasis suppressor miRNA in breast cancer [241]. On the other hand, microarray analysis of BrM CRC tumors revealed that miR-126 is upregulated in BrM CRC [132]. 

## 5. miRNA Based Therapeutic Oligonucleotides

**Figure 3:**
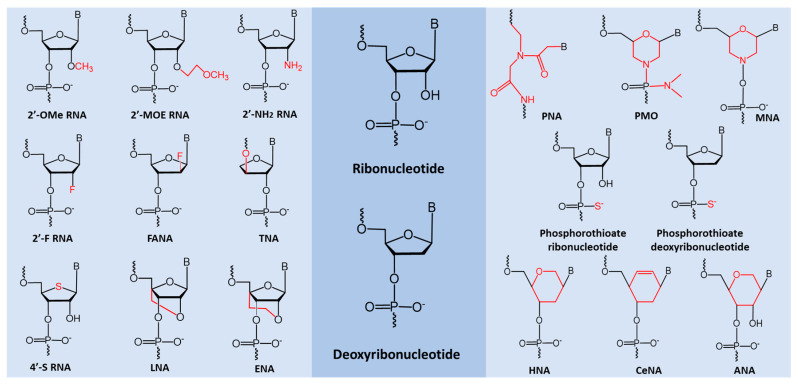
Examples of nucleotide analogues used in oligonucleotide synthesis. 2′-OMe, 2′-O-methyl [250,251]; 2′-MOE, 2′-O-methoxyethyl [252]; 2′-NH_2,_ 2′-amino [253]; 2′-F, 2′-fluoro [254]; 2′-FANA, 2′-fluroarabino nucleic acid [255]; TNA, threose nucleic acid [256]; 4′-S, 4′-thio [257]; LNA, locked nucleic acid [258,259,260]; ENA, 2′-O, 4′-C-ethylene-bridged nucleic acid [261]; PNA, peptide nucleic acid [262]; PMO, phosphorodiamidate morpholino oligomer [263]; MNA, morpholino nucleic acid [264]; Phosphorothioate, PS [265]; HNA, 1,5-anhydro hexitol nucleic acid [266]; CeNA, cyclohexenyl nucleic acid [266]; ANA, altritol nucleic acid [266].

Given the fact that a single miRNA can act as a tumor suppressor (oncogene inhibitor) in one context, as well as an oncomiR that inhibits expression of tumor suppressor genes in another context [242], miRNA-based therapeutic oligonucleotides can be developed accordingly. Specifically, mimics of tumor suppressor miRNAs and anti-miRNAs (antisense oligonucleotides) can be designed. The former miRNA mimics imitate the function of tumor suppressor miRNAs in an attempt to inhibit tumor development [243,244,245], while the latter (anti-miRNAs) are used to bind to oncomiR in order to deactivate their function through a steric blockage mechanism [242,246,247,248]. Importantly, therapeutic oligonucleotides composed of naturally occurring nucleotides (i.e., deoxyribonucleotide or ribonucleotide) are not suitable for clinical applications as they are easily degraded by nucleases in vivo, lack binding affinity and specificity. Therefore, chemically modified nucleotide analogues are introduced into the synthesis of oligonucleotides, which greatly improve their drug-like properties in terms of nuclease stability, binding affinity, and selectivity. Examples of sugar-modified nucleotide analogues [249] used to improve the oligonucleotide synthesis are provided in Figure 3. 

## 6. Conclusions and Prospects

**Figure 4:**
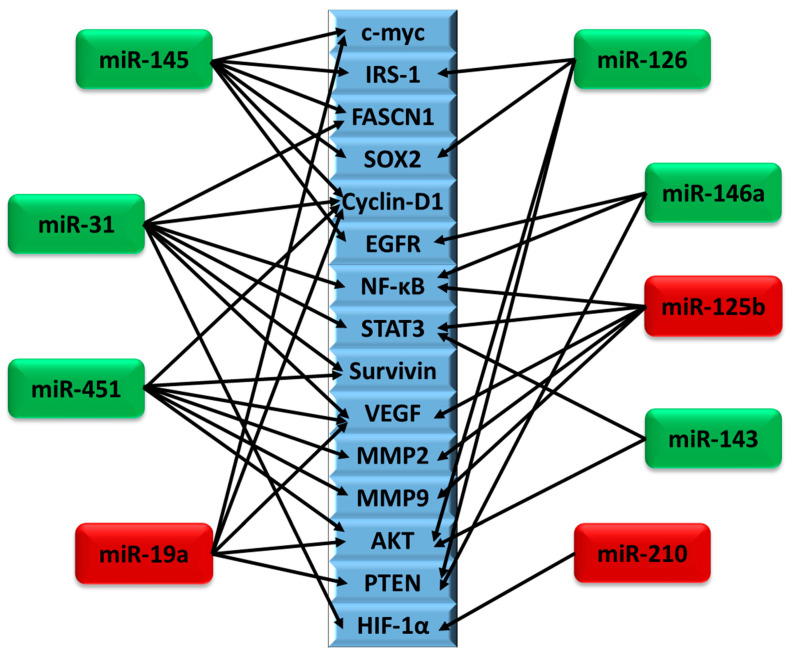
Schematic representation of genes regulated by multiple miRNAs in GBM and BrM. Among the genes that are reported to be regulated by the miRNAs reviewed above, the most common genes that are regulated by at least two miRNAs are represented (blue rectangles). The arrows indicate interactions between miRNAs and their targets. miRNAs indicated in green and red are predominantly tumor suppressors and oncomiRs, respectively. Expression of Cyclin-D1, VEGF, and AKT, which plays a major role in cell proliferation, angiogenesis, and metastasis, was shown to be modulated by four different miRNAs. Although miR-328 is reviewed above, it did not share any common targets with other miRNAs.

MicroRNA-based therapeutics are gaining importance due to their multi-gene targeting properties. Based on the expression of target proteins in a given tumor microenvironment, miRNAs could post-transcriptionally upregulate or downregulate proteins that play a significant role in cancer. It is evident that oncogenicity and tumor suppressive roles of miRNAs vary between different tumor types and occasionally within the same tumor. Figure 4 shows some of the genes reported to be regulated by multiple miRNAs. They play an important role in cell proliferation, apoptosis, and stemness, which modulate tumor growth and metastasis. Most of these miRNAs were reported to regulate proteins involved in major pathways including but not limited to PI3K/Akt, Wnt/β-catenin, NF-κB, and Notch signaling pathways, thereby suppressing or enhancing tumor growth. Among the ten miRNAs discussed here, some of them are predominantly reported to be tumor suppressors (miR-145, miR-31, miR-451, miR-143, miR-146a, miR-126) and oncomiRs (miR-19a, miR-125b, miR-210). Mimics of tumor suppressor miRNAs including miR-145, miR-31, miR-451, miR-143, miR-146a, and miR-126 could serve as potential therapeutic molecules for tackling primary and metastatic brain cancers. In addition, antimiRs (or antagomiRs) of miR-19a, miR-125b, and miR-210 could be used to suppress glioblastoma progression and metastasis. It should be noted that the primary cancer site should be taken into consideration while adapting miRNA-based therapeutics for metastatic brain cancers. miRNAs such as miR-145, miR-31, miR-125b, and miR-126 could be considered as prognostic markers in GBM progression. Targeting these differentially regulated miRNAs in BrM would improve the efficiency of therapies.

Miravirsen (SPC3649) was the first antimiR drug candidate to enter phase 2 clinical trials [267,268,269], and demonstrated the impact of miRNA targeting approach. Miravirsen is a 15bp locked nucleic acid (LNA) antisense oligonucleotide targeting miR-122, which showed to be effective for the treatment of hepatitis C [268]. RGLS5579 (Regulus therapeutics) is an antimiR targeting miR-10b, that showed an increased survival rate in GBM animal models as a monotherapy and in combination with temozolomide. This drug is now considered for clinical trials in GBM patients [270]. Due to the complexity and number of miRNA interactions, and dysregulation of multiple pathways within cancers, it is unlikely that modulating the expression of a single miRNA will have a sustained effect on the overall phenotype. On account of possible compensatory effects amongst different miRNAs, an approach looking at the whole regulatory network would be beneficial to evaluate potential off target effects and for developing better therapeutic outcomes.

## Figures and Tables

**Table 1:** Results of Venn diagram-based comparison of miRNAs differentially expressed in GBM and BrM. Upregulation and downregulation are abbreviated by ‘Up’ and ‘Down’, respectively.

Names	Total	miRNAs
BrM-down BrM-up GBM-down GBM-up	1	miR-145
BrM-down GBM-down GBM-up	2	miR-31 miR-451
BrM-up GBM-down GBM-up	5	miR-19a miR-143 miR-125b miR-328 miR-210
BrM-down BrM-up GBM-down	2	miR-146a miR-126
GBM-down GBM-up	31	miR-221/222 miR-200b miR-132 miR-185 miR-100 miR-135b miR-329 miR-106a miR-873 miR-205 miR-23a miR-323 miR-26b miR-200a miR-30bc miR-330 miR-146 miR-29b miR-9 miR-16 miR-15b miR-195 miR-130a miR-25 miR-23b miR-296 miR-141 miR-29a miR-27a miR-26a miR-107
BrM-down GBM-down	1	miR-7
BrM-up GBM-down	5	miR-197 miR-133a miR-378 miR-184 miR-133b
BrM-down GBM-up	1	miR-95
BrM-up GBM-up	2	miR-21 miR-10b
GBM-down	61	miR-497 miR-429 miR-137 miR-181d miR-129 miR-153 miR-487b miR-340 miR-150 miR-320 miR-339-5p miR-34a miR-491 miR-520b miR-331 miR-101 miR-410 miR-432 miR-181b miR-149 miR-377 miR-190 miR-422a miR-203 miR-124 miR-299 miR-136 miR-485 miR-610 miR-152 miR-146b-5p miR-634 miR-139 miR-219 miR-297 miR-513 LET-7 miR-365a miR-548b miR-181a miR-511-1 miR-218 miR-32 miR-128a miR-885 miR-154 miR-326 miR-433 miR-187 miR-379 miR-519a miR-483 miR-181 miR-874 miR-181c miR-138 miR-663 miR-182 miR-125a miR-148 miR-128
GBM-up	51	miR-142 miR-204 miR-92a miR-155 miR-20a miR-425 miR-519d miR-28 miR-182/183 miR-10a miR-193 miR-23 miR-93 miR-595 miR-24 miR-140 miR-30a miR-196 miR-148a miR-15a miR-215 miR-96 miR-655 miR-455 miR-196ab miR-130b miR-371-373 miR-18a miR-19b miR-92 miR-486 miR-27b miR-200c miR-301a miR-363 miR-383 miR-134 miR-92b miR-106b miR-123 miR-381 miR-17 miR-27 miR-367-302 miR-339 miR-603 miR-335 miR-372 miR-516-3p miR-17-92 cluster miR-582-5p
BrM-down	6	HS-170 miR-509 miR-1258 miR-30c miR-768-3p miR-29c
BrM-up	9	miR-576-5p miR-1 miR-199b HS-287 miR-200 miR-330-3p miR-28-5p miR-22 miR-199a
